# Adherence to personal protective equipment use among healthcare workers caring for confirmed COVID-19 and alleged non-COVID-19 patients

**DOI:** 10.1186/s13756-020-00864-w

**Published:** 2020-12-10

**Authors:** Meike M. Neuwirth, Frauke Mattner, Robin Otchwemah

**Affiliations:** 1 Institute for Hygiene, Cologne Merheim Medical Centre, University Hospital Witten/Herdecke Cologne, Ostmerheimer Str. 200, 51109 Cologne, Germany; 2 Chair of Hygiene and Environmental Medicine, Faculty of Health/Department of Human Medicine, University Witten/Herdecke, Ostmerheimer Str. 200, 51109 Cologne, Germany; 3 Department of Trauma and Orthopaedic Surgery, Cologne Merheim Medical Centre, University Hospital Witten/Herdecke Cologne, Ostmerheimer Str. 200, 51109 Cologne, Germany

**Keywords:** Coronavirus disease 2019 (COVID-19), Personal protective equipment (PPE), Adherence, Surgical face mask (SFM), FFP2-mask, Health care worker (HCW)

## Abstract

Adherence observations of health care workers (HCW) revealed deficiencies in the use of recommended personal protective equipment (PPE) among HCW caring in COVID-19 and non-COVID-19 wards during the first period of the SARS-CoV-2 pandemic in a university hospital in Germany. The adherence to wearing surgical face or FFP2-masks and disinfecting hands prior to donning and after doffing the PPE was significantly higher in COVID-19 wards However, there was no total adherence of 100% in COVID-19 wards.

## Introduction

The coronavirus disease 2019 (COVID-19) pandemic is a burden on societies and health care systems worldwide.

Regarding COVID-19 patient outcomes, medical risk factors and the capacity of health systems, especially the availability of well-trained health care workers (HCW), are decisive factors [1]. In Wuhan, staff shortage was found to be a major factor for the increased mortality rates [2], which was mainly due to COVID-19 infections among medical personnel acquired during patient care [2]. Particularly, if HCW were pre-symptomatic or asymptomatic carriers, they might have contributed to additional transmissions [3]. Therefore, protecting HCW from infection with SARS-CoV-2 is an important factor in controlling the SARS-CoV-2 epidemic [4].

According to current knowledge, SARS-CoV-2 is thought to be transmitted via droplets or aerosols during close, unprotected contacts or by direct and indirect contact [4].

Since a vaccine or treatment is still lacking, current SARS-CoV-2 prevention measures aim to interrupt transmissions by maintaining adequate hand hygiene and the use of personal protective equipment (PPE) consisting of protective gowns, gloves, surgical face masks (SFM) or filtering face pieces (FFP2) and goggles or visors as indicated. However, PPE have not been always available, were not worn or worn incorrectly, and mistakes during donning and doffing were documented [5, 6]. In a Study by Phan et al. [5] it was observed that 90% of doffing processes were incorrect. The most common errors occurred in the aspect of the correct removal of gowns (65%) and contact with potentially contaminated surfaces (48%) [5]. Ran et al. [6] reported that a lack of hand hygiene after contact with COVID-19 patients led to a higher risk of COVID-19 in Wuhan. For this reason, deficits in the use of PPE are to be identified and analyzed in order to provide HCW with targeted training on the correct and indication-appropriate use of PPE. In this context it is assumed that the general use of PPE has weaknesses and that HCW who are more experienced with respiratory and COVID-19 infections perform better than those who are inexperienced.

Here, we investigated the adherence to PPE use in COVID-19 and non-COVID-19 wards during the first epidemic phase of SARS-CoV-2 in a German university hospital.

## Methods

A prospective observational study was conducted in eight wards (two intensive-, two intermediate-, and four standard care units) at a university hospital in Cologne/Germany from February 27 to April 21, 2020. One intensive, one intermediate, and one standard care unit, all belonging to the Pulmonology Department and already experienced with infectious respiratory diseases before the COVID-19 pandemic, were exclusively dedicated to COVID-19 patients (hereinafter referred to as COVID-19 wards). Staff on these wards treated patients with respiratory infections on a regular basis and therefore frequently use the required PPE items*.* The remaining wards on which no COVID-19 patients were treated were called non-COVID-19 wards.

Based on national recommendations, a checklist of 18 items was compiled [4] (Additional file 1). It contained items to assess the processes of donning, wearing, and doffing of the PPE with the necessary work steps such as the required hand disinfection (HD). In COVID-19 wards, FFP2-masks had to be worn (only in rooms with SARS-CoV-2 positive patients). Adherence to the single and total process steps of donning and doffing of all the observed situations was calculated as the number of “yes” answers divided by sum of the number of “yes” and “no” answers. If an activity was carried out incorrectly, it was considered as “no”. Adherence is considered sufficient if the percentage value is greater or equal to 80%. The observation results for the indication “no wearing of jewelry on hands and wrists” were inverted for the analysis due to their negative formulation.

Observations were performed by trained infection control nurses during patient care in the context of hand hygiene compliance observations, which are anchored in the legal requirements of the German Protection against Infection Act (§23 IfSG) and retrospectively evaluated. The correct wearing and fit of PPE were evaluated by the trained observer based on national recommendations [4]. The observed HCW were aware of and agreed with the observation situations. The observations were made openly. At the beginning of the COVID-19 pandemic, a hygiene plan was developed and all HCW were trained on the use of the necessary PPE before the observations. In addition, a general obligation to wear SFM was introduced throughout the hospital. PPE had to be worn when treating COVID-19 patients as well as when treating patients with other infectious diseases, such as multi-resistant pathogens [7]. At no time during the observation period was there a lack of protective equipment at the observing COVID-19 and non-COVID19 wards.

Chi-square test was used as appropriate. The Chi-square test could only be calculated if the expected cell frequencies of one or more cells were greater than 5. The Phi value was calculated as a measure of the effect strength.

## Results

During the study period, 127 situations requiring PPE were observed in 87 nurses, 22 physicians, and 18 other employees (93 females; 34 males) (several multiple observations).

A total of 79 observations [intensive (*N* = 40), intermediate (*N* = 38), standard care units (*N* = 1)], which included 776 process steps, were performed on COVID-19 wards and 47 observations [intensive (*N* = 18), intermediate (*N* = 6), standard care units (*N* = 23)] with 410 process steps on non-COVID-19 wards.

The results of the observations showed a significantly higher overall adherence for COVID-19 wards experienced with respiratory tract infections compared to non-COVID-19 wards, especially with regard to hand hygiene and donning of PPE (Table 1).

**Table 1:** Comparison of the adherence rates of the indications for the use of protective equipment by Healthcare workers in COVID-19 and non-COVID-19 wards

	Indications/process steps	HCW in COVID-19 wards		HCW in non-COVID-19 wards		*p*^*b*^	*φ*^*c*^
		Adherence	*N*	Adherence	*N*		
Hand hygiene	No wearing of jewelry on hands and wrists	99%	79	69%	48	< .001***	.438
	HD before donning PPE	85%	59	54%	41	.001***	− .341
	HD at the end of the doffing of gowns and gloves	80%	59	81%	32	.856	.019
	HD after doffing eye protection^b^	57%	37	66%	3		
	final HD at the end of the doffing process	91%	65	54%	35	< .001***	− .420
	Total adherence to hand hygiene	82%	299	65%	159	< .001***	− .243
Donning	Correct donning of SFM and FFP2	89%	47	70%	47	.021*	− .238
	Correct fit of SFM and FFP2 and additional fit test of FFP2	38%	50	5%	43	< .001***	− .398
	Correct protective gown donning	91%	66	94%	35	.550	.059
	Donning eye protection^b^	84%	43	100%	2		
	Donning protective gloves	93%	72	97%	33	.422	.078
	Total adherence to donning	79%	278	73%	160	< .001***	− .186
Doffing	Wipe disinfection of the work surface^a^	79%	24	Desinfection was not required	0		
	Doffing gowns and gloves without self-contamination and without environmental contamination	88%	67	91%	32	.704	.038
	Doffing eye protection^b^	94%	36	100%	2		
	Correct doffing of SFM and FFP2	96%	48	80%	25	.029*	− .255
	Disposal of the materials in correct waste^a^	100%	48	100%	32		
	Total adherence to doffing^e^	95%	199	93%	91	.389	− .051
Total adherence to PPE use		85%	776	76%	410	< .001***	− .109

^a^Significance level could not be calculated

^b^Chi-square test could not be calculated because expected cell frequencies of one or more cells were less than 5

^c^*p ≤ .05 (significant), **p ≤ 01 (highly significant), ***p ≤ .001 (highly significant)

^d^φ (Phi) ≤ .10 (small effect), φ = .30 (moderate effect), φ ≥ .50 (large effect)

^e^The indication “wipe disinfection of the work surface” was not considered in the calculation, as it was not required for non-COVID-19 wards

**Fig. 1:**
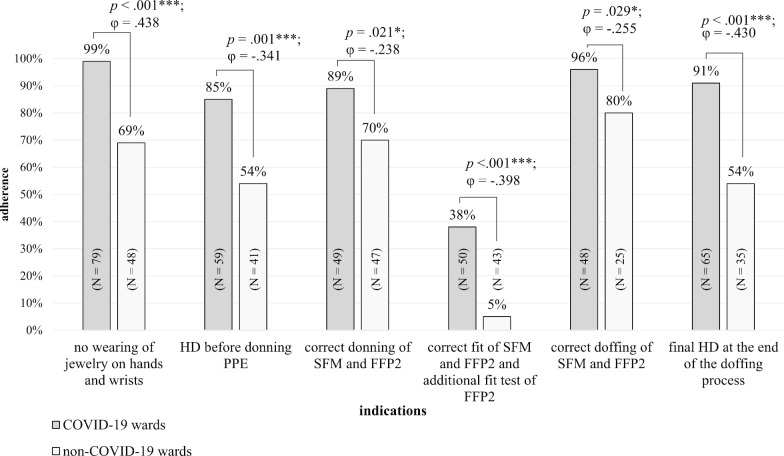
Differences in adherence regarding PPE use in COVID-19 and non-COVID-19 wards

On the level of the individual process steps with regard to the indications of hand hygiene, COVID-19 wards showed significantly higher adherence rates for the indications “no wearing of jewelry on hands and wrists”, “HD before donning PPE”, and “final HD at the end of the doffing process” (Table 1 and Fig. 1).

For the donning of PPE, significantly higher adherence rates were also found in the COVID-19 wards for the indications “correct donning of SFM/FFP2”, “correct removal of SFM/FFP2”, “final HD”, and “correct fit of SFM and FFP2 and additionally fit test of FFP2” (Fig. 1 and Table 1). Thereby, the indication “correct fit of SFM and FFP2 and additionally fit test of FFP2” showed the lowest adherence overall (Fig. 1 and Table 1).

For the doffing of PSA, no significant difference in total adherence between COVID-19 and non-COVID-19 wards could be observed. The COVID-19 wards showed a significantly higher adherence rate only for the single indication “correct doffing of SFM/ FFP2 masks”.

The results of all observed indications are shown in Table 1.

## Discussion

Generally, the COVID-19 wards showed a higher total adherence with 85% of PPE use compared to the non-COVID-19 wards with a total adherence of 76%. Particularly, the increased adherence in the areas of hand hygiene and wearing PPE had a major impact on the overall adherence. For PSA doffing, there was no significant difference in adherence between COVID-19 (95%) and non-COVID-19 wards (93%) when removing the PPE.

The hand hygiene adherence of HCWs in the COVID-19 wards was performed clearly above the national standard (median of 79% for all indications on intensive care units) [8] appropriate for a response to the pandemic challenge of SARS-CoV-2.

The adherence to the different recommendations “no wearing of jewelry on the hands and wrists”, “HD before donning PPE”, and “final HD after patient care” was significantly lower among HCW in non-COVID-19 compared to COVID-19 wards. The execution of HD in the process of doffing PPE, especially at the end of the process, is necessary in order not to contaminate oneself with pathogens [9]. In Wuhan, it was shown that a lack of hand hygiene increased the risk of transmitting SARS-CoV-2 from patients to HCW after hand contamination [6].

Although the COVID-19 wards performed better, we were surprised to detect deficits in fitting the masks (either SFM or FFP2) in a high proportion of all the observed wards. A leakage, especially by FFP2 masks and the incorrect wearing of SFM e.g. wearing the mask under the nose, could scotch any preventive effect. Probably the knowledge of the details on how to wear a mask correctly and the exercise on how to wear it in routine practice is still lacking. Our observation shows similar results to a quantitative fit test compliance study in which 38.2% of subjects failed the test [10].

Deficits in the everyday handling of PPE have been observed before (especially in fitting, considering the correct sequence and correct use) and were found in 90% of the personnel [5]. The most common errors occurred in the correct removal of gowns (65%) and contact with potentially contaminated surfaces (48%) [5].

A reason for better hand hygiene adherence and performance in donning and doffing protective equipment could be due to the greater experience of the COVID-19 wards in dealing with respiratory tract diseases and PPE. In addition, increased situation-related higher awareness and risk awareness could also be a reason for better adherence in handling PPE.

In summary, we observed deficits in PPE use among all observed HCWs. Experienced HCWs showed higher adherence in the use of PPE than less experienced ones. However, despite the high awareness of the HCW regarding the dangers of SARS-CoV-2, it is surprising that they could not adhere to the fitting of FFP2-masks in COVID-19 and SFM in non-COVID-19 wards in which undetected SARS-CoV-2-positive patients or HCW might have been present at time. Thus, there is still a clear need for training in the correct and indication-appropriate use of PPE in general and wearing masks in particular, to protect HCW from infection by droplet or even aerosol transmissible pathogens.

## Supplementary Information

**Additional file 1** Checklist for PPE observation of HCW in COVID-19 and non-COVID-19 wards.

## Data Availability

Not applicable.
