# Supplementary material for: Adherence to personal protective equipment use among healthcare workers caring for confirmed COVID-19 and alleged non-COVID-19 patients
Source: Antimicrob Resist Infect Control. 2020 Dec 10;9:199. doi: 10.1186/s13756-020-00864-w (PMC7726598; doi:10.1186/s13756-020-00864-w)
Supplement: Supplementary file 1 — Additional file 1 Checklist for PPE observation of HCW in COVID-19 and non-COVID-19 wards. [file 13756_2020_864_MOESM1_ESM.pdf]

Checkliste CoV-2 (sheet for 7 observations) sheet-no.: _________ observer-ID: _________ date:_____________________

		observation-no.:	observation-no.:	observation-no.:	observation-no.:	observation-no.:	observation-no.:	observation-no..:
	Indication donning	professional group:<br>female ☐ male ☐<br>ward:	professional group:<br>female ☐ male ☐<br>ward:	professional group:<br>female ☐ male ☐<br>ward:	professional group:<br>female ☐ male ☐<br>ward:	professional group:<br>female ☐ male ☐<br>ward:	professional group:<br>female ☐ male ☐<br>ward:	professional group:<br>female ☐ male ☐<br>ward:
donning	no wearing of jewelry on hands and wrists	☐ no<br>☐ yes	☐ no<br>☐ yes	☐ no<br>☐ yes	☐ no<br>☐ yes	☐ no<br>☐ yes	☐ no<br>☐ yes	☐ no<br>☐ yes
	hand disinfection before donning PPE	☐ no<br>☐ yes	☐ no<br>☐ yes	☐ no<br>☐ yes	☐ no<br>☐ yes	☐ no<br>☐ yes	☐ no<br>☐ yes	☐ no<br>☐ yes
	correct protective gown donning	☐ no<br>☐ yes	☐ no<br>☐ yes	☐ no<br>☐ yes	☐ no<br>☐ yes	☐ no<br>☐ yes	☐ no<br>☐ yes	☐ no<br>☐ yes
	correct donning of SFM/FFP2 [a]	☐ no<br>☐ yes, SFM<br>☐ yes, FFP2	☐ no<br>☐ yes, SFM<br>☐ yes, FFP2	☐ no<br>☐ yes, SFM<br>☐ yes, FFP2	☐ no<br>☐ yes, SFM<br>☐ yes, FFP2	☐ no<br>☐ yes, SFM<br>☐ yes, FFP2	☐ no<br>☐ yes, SFM<br>☐ yes, FFP2	☐ no<br>☐ yes, SFM<br>☐ yes, FFP2
	correct fit of SFM/FFP2 and additional fit test of FFP2 [b]	☐ no<br>☐ yes	☐ no<br>☐ yes	☐ no<br>☐ yes	☐ no<br>☐ yes	☐ no<br>☐ yes	☐ no<br>☐ yes	☐ no<br>☐ yes
	donning eye protection	☐ no<br>☐ yes, goggles<br>☐ yes, visor	☐ no<br>☐ yes, goggles<br>☐ yes, visor	☐ no<br>☐ yes, goggles<br>☐ yes, visor	☐ no<br>☐ yes, goggles<br>☐ yes, visor	☐ no<br>☐ yes, goggles<br>☐ yes, visor	☐ no<br>☐ yes, goggles<br>☐ yes, visor	☐ no<br>☐ yes, goggles<br>☐ yes, visor
	donning protective gloves	☐ no<br>☐ yes	☐ no<br>☐ yes	☐ no<br>☐ yes	☐ no<br>☐ yes	☐ no<br>☐ yes	☐ no<br>☐ yes	☐ no<br>☐ yes

	Indication doffing	observation-no.:	observation-no.:	observation-no.:	observation-no.:	observation-no.:	observation-no.:	observation-no..:
doffing	doffing gowns and gloves without self-contamination and without environmental contamination	☐ no<br>☐ yes	☐ no<br>☐ yes	☐ no<br>☐ yes	☐ no<br>☐ yes	☐ no<br>☐ yes	☐ no<br>☐ yes	☐ no<br>☐ yes
	hand disinfection at the end of the doffing of gowns and gloves	☐ no<br>☐ yes	☐ no<br>☐ yes	☐ no<br>☐ yes	☐ no<br>☐ yes	☐ no<br>☐ yes	☐ no<br>☐ yes	☐ no<br>☐ yes
	doffing eye protection	☐ no<br>☐ yes	☐ no<br>☐ yes	☐ no<br>☐ yes	☐ no<br>☐ yes	☐ no<br>☐ yes	☐ no<br>☐ yes	☐ no<br>☐ yes
	hand disinfection after doffing eye protection	☐ no<br>☐ yes	☐ no<br>☐ yes	☐ no<br>☐ yes	☐ no<br>☐ yes	☐ no<br>☐ yes	☐ no<br>☐ yes	☐ no<br>☐ yes
	correct doffing of SFM/FFP2	☐ no<br>☐ yes	☐ no<br>☐ yes	☐ no<br>☐ yes	☐ no<br>☐ yes	☐ no<br>☐ yes	☐ no<br>☐ yes	☐ no<br>☐ yes
	hand disinfection after doffing of SFM/FFP2	☐ no<br>☐ yes	☐ no<br>☐ yes	☐ no<br>☐ yes	☐ no<br>☐ yes	☐ no<br>☐ yes	☐ no<br>☐ yes	☐ no<br>☐ yes
	disposal of the materials in correct waste and cleaning for reusable eye protection/face visor	☐ no<br>☐ yes<br>☐ not required	☐ no<br>☐ yes<br>☐ not required	☐ no<br>☐ yes<br>☐ not required	☐ no<br>☐ yes<br>☐ not required	☐ no<br>☐ yes<br>☐ not required	☐ no<br>☐ yes<br>☐ not required	☐ no<br>☐ yes<br>☐ not required
	wipe disinfection of the work surface	☐ no<br>☐ yes<br>☐ not required	☐ no<br>☐ yes<br>☐ not required	☐ no<br>☐ yes<br>☐ not required	☐ no<br>☐ yes<br>☐ not required	☐ no<br>☐ yes<br>☐ not required	☐ no<br>☐ yes<br>☐ not required	☐ no<br>☐ yes<br>☐ not required
	Final hand disinfection at the end of the doffing process	☐ no<br>☐ yes	☐ no<br>☐ yes	☐ no<br>☐ yes	☐ no<br>☐ yes	☐ no<br>☐ yes	☐ no<br>☐ yes	☐ no<br>☐ yes
	remarks							

a) The indication of correct donning of SFM/FFP2 included the attachment of SFM/FFP2 via rubber bands and the avoidance of touching the inside and front of a mask as well at the spreading of the mask up to the chin.
b) The indication correct fit of SFM/FFP2 includes the modifying of the nose bow. In non-COVID-19 wards any contact to patients required wearing a SFM. In COVID-19 wards FFP2-mask had to be worn (only in rooms with SARS-CoV-2 positive patients).
